# Protective efficacy of intranasal inactivated pseudorabies vaccine is improved by combination adjuvant in mice

**DOI:** 10.3389/fmicb.2022.976220

**Published:** 2022-09-15

**Authors:** Tao Hua, Chen Chang, Xuehua Zhang, Yuqing Huang, Haiyan Wang, Daohua Zhang, Bo Tang

**Affiliations:** ^1^Institute of Veterinary Immunology & Engineering, Jiangsu Academy of Agricultural Sciences, Nanjing, China; ^2^National Research Center of Veterinary Bio-product Engineering and Technology, Jiangsu Academy of Agricultural Science, Nanjing, China; ^3^Jiangsu Key Laboratory for Food Quality and Safety-State Key Laboratory Cultivation Base, Ministry of Science and Technology, Nanjing, China; ^4^Jiangsu Co-innovation Center for Prevention and Control of Important Animal Infectious Diseases and Zoonoses, Yangzhou, China

**Keywords:** pseudorabies virus, intranasal vaccine, mucosal immunity, Gel 01, CVCVA5, combination adjuvant, protective efficacy

## Abstract

Pseudorabies virus (PRV) not only causes great economic loss to the pig industry but also seriously threatens the biosafety of other mammals, including humans. Since 2011, PRV mutant strains have emerged widely in China, and the classical Bartha-K61 vaccine cannot confer complete protection for pigs. PRV mainly infects pigs *via* the respiratory tract. Intranasal immunization with PRV has received more attention because intranasal vaccination elicits systemic and mucosal immune responses. To induce systemic and mucosal immune responses against PRV, we developed a combination adjuvant as a delivery system for intranasal vaccine, which was formulated with MONTANIDE™ Gel 01 and CVCVA5. In comparison to naked antigen of inactivated PRV, single Gel 01 adjuvanted inactivated antigen and single CVCVA5 adjuvanted inactivated antigen, intranasal inactivated PRV vaccine formulated with the combination adjuvant induced greater mucosal IgA immunity and serum antibody responses (IgG, IgG1, and IgG2a). Furthermore, the production of the Th1-type cytokine IFN-γ and the Th2-type cytokine IL-4 indicated that the cellular and humoral responses to the intranasal vaccine were improved by the combination adjuvant. In addition, the intranasal vaccine formulated with the combination adjuvant induced long-term T lymphocyte memory with increased central (CD62L^+^CD44^+^) and effector (CD62L^–^CD44^+^) memory subsets of both CD4 and CD8 T cells in nasal-associated lymphoid tissue. Intranasal challenge with virulent PRV in mice showed that the protective efficacy of the intranasal PRV vaccine was improved by the combination adjuvant compared with the other single-adjuvanted vaccines. In summary, these data demonstrated that Gel 01 combined with the CVCVA5 adjuvant induced a synergistic effect to improve mucosal immunity and protective efficacy of the intranasally inactivated PRV vaccine in mice. It represents a promising vaccination approach against PRV infection.

## Introduction

Pseudorabies (PR), or Aujeszky’s disease, is caused by pseudorabies virus (PRV). PRV belongs to the family Herpesviridae, subfamily Alphaherpesvirinae, and genus Varicellovirus (Mettenleiter, 2000). It is a serious swine pathogen that can cause fatal encephalitis in newborn pigs, respiratory disorders in growing-fattening pigs, and reproductive failure in sows, leading to great economic loss worldwide (Szpara et al., 2011). PRV always directly causes lethal infection in other species, such as sheep, cattle and mice (Kong et al., 2013). Mice are often used as model animals to evaluate PRV virulence and PRV vaccine protection due to their convenience and standardization (Nakamura et al., 1993; Brittle et al., 2004; Yu et al., 2017; Wang et al., 2019). Many types of PRV vaccines have played important roles in controlling PR over the years (Freuling et al., 2017). However, since late 2011, novel antigenic variant PRV isolates have emerged in many pig herds immunized with conventional PRV vaccines and spread widely to most pig farms in China (Tan et al., 2021). Conventional vaccines could not provide sufficient protection against the newly emerging PRV variants. The new PRV variants cause severe economic loss to the Chinese swine industry and threaten the world’s biosecurity (Sun et al., 2016).

The most common pathway of PRV infection is through the mucosa of the upper respiratory tract. When PRV transmits through the nasal cavity, the virus primarily replicates in epithelial cells of the surface mucosa of the upper respiratory tract before attacking sensory nerve endings, crossing synapses to infect neurons and invading the nervous system (Bosch et al., 2013; Lamote et al., 2016). Therefore, intranasal immunization might be an ideal measure against PRV infection. Initial reports showed that intranasal vaccination of pigs using attenuated live vaccines conferred good protection (van Oirschot and Gielkens, 1984; Van Oirschot, 1987; Nauwynck et al., 1999). Attenuated live viruses remain alive, and reversion to virulence or interactions with other pathogens present at the time of inoculation have been reported (Liu et al., 2018; Eclercy et al., 2019). Traditional attenuated PRV also has some security problems, resulting in the spread of PRV across different species (Li et al., 2017). Epidemiological data analysis proved that PRV has the potential to infect humans (Wong et al., 2019). The interspecies transmission mechanism and evolutionary dynamics of PRV also indicated the potential risk of PRV transmission between humans and animals (He et al., 2019). Thus, it is necessary to develop a more efficacious and safe vaccine to control virulent PRV variants.

Intranasal vaccination with inactivated vaccines displays high safety for vaccinated animals without viral virulence reversion, while these vaccines are generally less efficacious compared to intranasal immunization with live attenuated and virus-vectored vaccines (Li et al., 2020). It is generally believed that intranasal inactivated vaccines require more effective adjuvants to overcome problems such as short residence time in the nose, rapid antigen clearance and immune tolerance, to enhance immunogenicity (Lycke, 2012). Mucoadhesive polymeric adjuvants have been widely used for mucosal antigen delivery because of their safety and ability to provide long-term controlled vaccine antigen release in mucosal immunized sites and to protect antigens from low pH, bile salts, and digestive enzymes (Lin et al., 2015). Many polymeric adjuvants, such as polylactic-co-glycolic acid, polyacrylate and hyaluronic acid, have been developed for vaccine delivery through the nasal cavity (Eyles et al., 1998; Zaman et al., 2010; Suzuki et al., 2022). Toll-like-receptors (TLRs) agonists are based on pathogen-associated molecular patterns and constitute a major category of mucosal adjuvants (Lycke, 2012). For example, muramyl dipeptide (MDP), which signals through NOD-like receptors, has also been used as an adjuvant in mucosal immunizations and has shown good potency (Shafique et al., 2013). Poly I:C-adjuvanted intranasal inactivated influenza vaccine induced cross-protective immunity against antigenic variant swine influenza viruses in pigs (Thomas et al., 2015). A combination adjuvant composed of polymeric nanoparticles and poly I:C significantly enhanced the immune response to an intranasal inactivated influenza vaccine in pigs (Renu et al., 2020). Combination adjuvants (AS03, AS04, and AS01B) have been evaluated as nasal mucosal vaccine adjuvants in animal models and in clinical trials (Xu et al., 2021).

MONTANIDE™ Gel 01 (SEPPIC, France) is a mucoadhesive polymeric adjuvant that can improve the safety and efficacy of mucosal vaccination (Deville et al., 2012; Li et al., 2019; Dessalegn et al., 2021). Gel 01 is based on a dispersion of highly stable gel particles of sodium polyacrylate in water. Polyacrylic acid and commercial derivatives (Carbopol^®^ and Carbomer^®^) have been reported and used as effective mucoadhesive and absorption-promoting agents (Gasper et al., 2016; Chen et al., 2019; Correia et al., 2022). The adjuvant CVCVA5 (VA5), which holds a Chinese patent license with the registered number 201210235427.0, improved the efficacy of both the serum and mucosal antibody response and the cell-mediated immune response of inactivated vaccine (Tang et al., 2014; Lu et al., 2016; Wu et al., 2017). The aqueous components of the VA5 adjuvant contain ligands for pattern recognition receptors, poly I:C, MDP, and a chemical with immune enhancement activity, levamisole hydrochloride. The poly I:C is the ligand of toll-like-receptors (TLRs)-3. The MDP is recognized by NOD-like receptors (NOD)-2. Levamisole is an antiparasitic agent and is also capable of immune enhancement (de-la-Rosa-Arana et al., 2012). In this study, we assessed the effect of a combination adjuvant composed of Gel 01 and VA5 on the mucosal immune response, systemic immune response, and protective efficacy of an intranasal inactivated PRV vaccine in mice. The effect of intranasally inactive PRV antigen formulated with a combination adjuvant of Gel 01 and VA5 was compared with naked PRV antigen, single Gel 01-adjuvanted PRV antigen, and single VA5-adjuvanted PRV antigen.

## Materials and methods

### Cells and virus

ST cells were purchased from the China Institute of Veterinary Drug Control (Beijing, China) and maintained in minimum essential medium (Gibco, Carlsbad, CA, United States) supplemented with 5% calf serum (Gibco, Carlsbad, CA, United States), 100 U/ml penicillin (Sigma-Aldrich, St. Louis, MO, United States) and 0.1 mg/ml streptomycin (Sigma-Aldrich, St. Louis, MO, United States). PRV strain SQ (3rd culture passage) was isolated in 2013 from a pig diagnosed with PR in Suqian city, Jiangsu Province, China. ST cells were placed in cell flasks at a density of 1 × 10^5^ cells/ml. After 24 h, the culture medium was removed, and fresh Dulbecco’s modified Eagle’s medium (Gibco, Carlsbad, CA, United States) containing 2% calf serum (Gibco, Carlsbad, CA, United States) was added and inoculated with PRV strain SQ at a multiplicity of infection of 0.001. After 80% of cells had a marked cytopathic effect, the cells and medium were harvested and stored at −80°C until use.

### Preparation of vaccines and adjuvants

For vaccine preparation, the cell culture fluid of PRV grown in ST cells was harvested and subjected to sucrose gradient ultracentrifugation. The virus pellet was suspended in phosphate-buffered saline (pH 7.2, PBS) and inactivated using β-propiolactone (v/v 0.5%, 24 h, 37°C, Sigma, St. Louis, MO, United States). Montanide™ Gel 01 was kindly provided by SEPPIC (Shanghai city, China). VA5 adjuvant was kindly provided by Associate Professor Lu Jihu (Jiangsu Academy of Agricultural Science). VA5 adjuvant was prepared in accordance with the procedures outlined in the Chinese patent (registration number: 201210235427.0, Supplementary Figure 1). Briefly, the components of VA5 adjuvant consist of an aqueous phase that contains L–D isoform muramyl dipeptide (MDP) (InvivoGen), poly I:C (InvivoGen) and levamisole hydrochloride (Sigma) dissolved in PBS (pH 7.2). One volume of VA5 or PBS was mixed with eight volumes of inactive PRV antigen. Nine volumes of inactive PRV antigen with or without VA5 were mixed with one volume of Gel 01 or PBS by shaking for 5 min. In a total volume of 20 μl, mice were intranasally immunized with 40 μg of PRV antigen formulated with or without VA5 (2 μl), Gel 01 (2 μl), or VA5 (2 μl) + Gel 01 (2 μl), respectively.

### Animal immunization and challenge

One hundred fifty healthy BALB/c female mice (5 weeks old) were purchased from the Experimental Animal Center of Yangzhou University and randomly divided into five groups (*n* = 30 per group). The A group was immunized with PBS, the B group was immunized with the inactive naked PRV antigen, the C group was immunized with the inactive PRV antigen formulated with Gel 01 polymer adjuvant, the D group was immunized with the inactive PRV antigen formulated with VA5 adjuvant, and the E group was immunized with the inactive PRV antigen formulated with the combination adjuvant comprised of VA5 and Gel 01. All formulations were delivered in a total volume of 20 μl, which was applied as droplets directly over both nares of the mice. At 21 days post-first immunization (dpi), all mice were boosted in the same manner. On Day 42, 5–8 mice from each group were sacrificed for cytokine assays in splenocytes and the analysis of T lymphocytes in nasal-associated lymphoid tissue (NALT). Six weeks after the first vaccination, mice (*n* = 10) were challenged intranasally with 10 × LD_50_ and 100 × LD_50_ of PRV strain SQ in 20 μl of PBS under anesthesia. After challenge, the mice were observed daily for 14 days for clinical signs of disease. All animal procedures were approved by the Science and Technology Agency of Jiangsu Province (approval number: NKYVET 2015-0066) and by the Jiangsu Academy of Agricultural Sciences Experimental Animal Ethics Committee. All animal studies were consistent with the guidelines outlined in the Jiangsu Province Animal Regulations (Government Decree No. 45).

### Serum and mucosal antibody titers by indirect ELISA

Antigen-specific serum antibodies (IgG total, IgG1, and IgG2a) and mucosal wash (nasal wash and lung wash) IgA antibodies were measured by ELISA. Briefly, PRV virus particles were purified by sucrose gradient ultracentrifugation and inactivated with β-propiolactone. The inactivated PRV virus particles were coated on polystyrene microtiter plates overnight at 4°C at a concentration of 5 μg PRV per well. Plates were blocked with 100 μl of DPBS plus 5% BSA for 1 h at 37°C, washed once with DPBS plus 1% BSA, and then incubated with 100 μl of serially diluted serum samples (1:100 to 1:12800 for IgG, IgG1, and IgG2a) and mucosal wash samples (1:20 to 1:2560 for IgA) for 1.5 h. After washing 5 times, the plates were incubated with 100 μl of a 1/10,000 dilution of HRP-conjugated goat anti-mouse IgG, IgG1, and IgG2a antibodies or 100 μl of a 1:2500 dilution of HRP-conjugated goat anti-mouse IgA antibodies for 1 h. The plates were washed 5 times and then incubated with 100 μl of the color substrate 3,3,5,5-tetramethyl benzidine (TMB) (Boshide, Wuhan, China) at room temperature for color development. After 15 min, the enzyme-substrate reaction was stopped by adding 50 μl of 2 M H_2_SO_4_ to each well. The optical density (OD) was read at 450 nm. At the same time, the sera and mucosal wash samples from the control group (PBS-treated) were used as negative controls. The titers were expressed as the highest dilution that resulted in an OD450 value greater than that of the mean + two standard deviations of negative for PRV.

### Flow cytometry

T cells from the NALT of immunized mice were isolated according to a previous report (Carrasco-Yepez et al., 2018). Briefly, blood was withdrawn by cardiac puncture of ether-anesthetized mice, and then they were sacrificed by cervical dislocation and decapitation. The nasal cavity of the immunized mouse was extracted by removing the brain, tongue, lower jaw, skins and muscle tissues to expose the soft palette of the upper jaw. The palate was then excised from the anterior end using a scalpel blade. After the incisions, the palate was gripped behind the incisor teeth with fine forceps and gently pulled toward the molar teeth while using the scalpel to gently release tissue among the palate, jawbones, and nasal septum. The palate was then cut into pieces and ground gently through a 70-μm sterile nylon net. The cell suspension was carefully collected, placed on RPMI-1640 medium (10% fetal bovine serum (FBS), 100 U/ml penicillin, and 0.1 mg/mL streptomycin), and washed 3 times. A total of 1 ml containing 1 × 10^6^ cells/well was cultured in 6-well plates. Sustained NALT cells were then cultured at 37°C for 6 h with 10 μg/ml of inactivated PRV. Subsequently, the cells were stained with the monoclonal antibodies anti-CD3 BV650, anti-CD4 BV786, anti-CD8 BUV396, anti-CD44 FITC, and anti-CD62L APC-Cy7 (BD Biosciences). The cells were fixed and permeabilized with Cytofix/Cytoperm (BD Biosciences). Finally, the samples were analyzed by a five-laser Fortesa X-20 flow cytometer (BD Biosciences).

### Cytokine assay

At 42 dpi, the mice were sacrificed by cervical dislocation under anesthesia, and the spleens were removed from the mice. The spleen was cut into pieces and ground gently through a 70-μm sterile nylon net. The cell suspension was carefully collected and placed on RPMI 1640 medium (10% FBS, 100 U/ml penicillin and 0.1 mg/ml streptomycin) and washed 3 times. One milliliter of splenocyte suspension containing 1 × 10^6^ cells/well was cultured in 6-well plates for 48 h, with 10 μg/ml inactivated PRV per well. Wells without antigen were set as the background controls, and wells with phorbol myristate acetate (PMA, Sigma, 100 ng/ml) were set as the positive controls. Cell supernatants were collected by centrifugation and stored at −80°C for cytokine analysis by ELISA kits according to the manufacturer’s protocols (Angle Gene Technologies, Nanjing, China).

### Statistical analysis

GraphPad Prism software, version 5.0 (San Diego, CA, United States), was used to perform statistical analyses. One-way analysis of variance and Tukey’s multiple comparison tests were used to analyze the significance of the difference between means. The data are expressed as the mean ± standard deviation. Statistical significance, set at *p* < 0.05, *p* < 0.01, and *p* < 0.001, indicated greater degrees of significance.

## Results

### Serum IgG responses to pseudorabies virus

**FIGURE 1:**
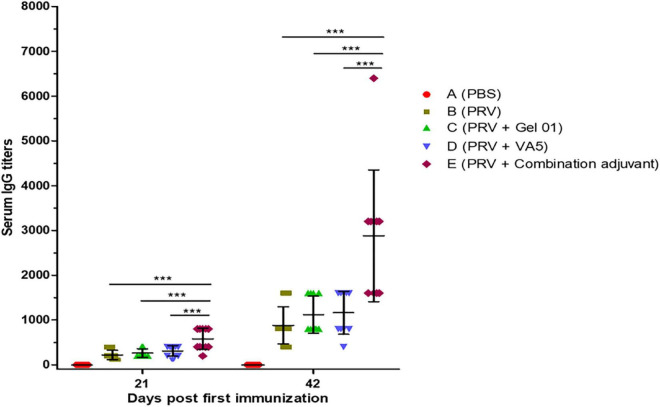
Pseudorabies virus (PRV)-specific IgG titers from mouse serum measured by ELISA at 21 and 42 dpi (*n* = 10). The results are shown as the mean ± SD. The asterisks indicate significant differences (***p < 0.001).

Serum IgG has been proven to generate immune defense not only in the systemic immune response but also in the lower respiratory tract (Renegar et al., 2004; Jearanaiwitayakul et al., 2021). To evaluate the impact of the intranasal inactivated PRV vaccine with different adjuvants on the systemic immune response, we examined PRV-specific total IgG in mice at 21 and 42 (Figure 1 and Supplementary Table 1). As shown in Figure 1, the results indicated that PRV-specific IgG significantly increased from 21 to 42 dpi. Meanwhile, the combination adjuvant composed of Gel 01 and VA5 (Group E) significantly promoted the IgG levels of intranasal vaccine compared with naked PRV antigen, single Gel 01 adjuvant and single VA5 adjuvant at 21 and 42 dpi (*p* < 0.001). However, Gel 01 (Group C) or VA5 (Group D) alone did not significantly raise PRV-specific IgG compared to naked PRV antigen (*p* > 0.05). These results demonstrate that the combination adjuvant comprising Gel 01 and VA5 efficiently boosts the systemic immune response of the intranasally inactivated PRV vaccine more so than a single Gel 01 adjuvant and a single VA5 adjuvant.

### Serum IgG1 and IgG2 responses against pseudorabies virus

**FIGURE 2:**
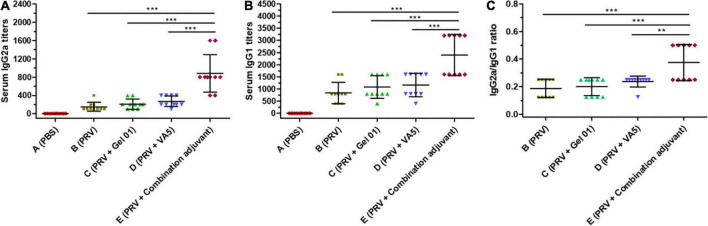
Antigen-specific IgG2a **(A)** and IgG1 **(B)** titers from mouse serum at 42 dpi (*n* = 10). The IgG1/IgG2a values of seropositive mice are displayed in **(C)**. The results are shown as the mean ± SD. The asterisks indicate significant differences (**p < 0.01, ***p < 0.001).

Th1-like immune responses included a multitude of IgG2a antibodies in serum, and Th2-like immune responses had numerous IgG1 antibodies (Firacative et al., 2018). The IgG isotypes IgG1 and IgG2a in sera were detected to investigate the influence of adjuvants on the quality of the immune response to the intranasal inactivated PRV vaccine (Figure 2 and Supplementary Table 2). As shown in Figures 2A,B, antigen-involved formulations elicited predominantly IgG1 responses (840 ± 440∼2400 ± 843 titers) in all vaccinated groups compared to lower IgG2a levels (150 ± 97∼880 ± 413 titers), which displayed an outstanding humoral response induced by inactivated antigen. The combination adjuvant composed of Gel 01 and VA5 (Group E) raised IgG2a 5.86-fold, 4.19-fold, and 3.25-fold (Figure 2A) and IgG1 2.85-fold, 2.22-fold, and 2.06-fold (Figure 2B) over naked PRV antigen, Gel 01 and VA5. However, Gel 01 (Group C) or VA5 (Group D) alone did not significantly increase IgG1 and IgG2a compared to naked PRV antigen (*p* > 0.05). In conclusion, a mixed enhancement of Th1/Th2 was elicited by antigen formulated with the combination adjuvant, which partially suggested that the combination adjuvant could amplify the mixed humoral and cellular responses.

The IgG2a/IgG1 ratios highlighted the Th1/Th2 polarization of the immune responses (Wu et al., 2012; Marciani, 2022). As displayed in Figure 2C, the combination adjuvant significantly increased the IgG2a/IgG1 ratios 2.00-fold, 1.87-fold, and 1.58-fold over naked PRV antigen, Gel01 and VA5, respectively (*p* < 0.01). However, neither Gel 01 nor VA5 significantly increased the IgG2a/IgG1 ratios compared with naked PRV antigen (*p* > 0.05). These results indicate that a compound adjuvant composed of Gel 01 and VA5 improves the quantity and quality of the immune response to intranasal vaccination.

### Mucosal IgA antibody response

**FIGURE 3:**
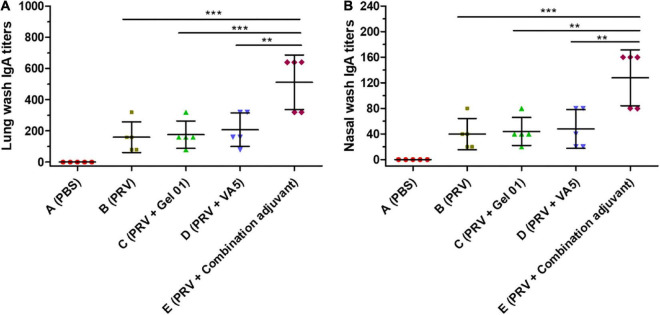
Mucosa IgA titers in lung wash **(A)** and nasal wash **(B)** at 42 dpi (*n* = 5). The results are shown as the mean ± SD. The asterisks indicate significant differences (**p < 0.01, ***p < 0.001).

The local production of secreted IgA (sIgA) antibodies is the most important characteristic that mediates nasal adaptive immunity and mucosal protection (Sterlin et al., 2021). The secretion of IgA against PRV was mainly detected in lung and nasal wash at 42 dpi (Figures 3A,B and Supplementary Table 3). The combination adjuvant (Group E) raised lung wash IgA 3.20-fold, 2.90-fold, and 2.46-fold (Figure 3A), and nasal wash IgA 3.20-fold, 2.90-fold, and 2.66-fold (Figure 3B) over naked PRV antigen, Gel 01 and VA5, respectively. The combination adjuvant (Group E) significantly induced a higher level of IgA in lung and nasal wash compared with the other groups (*p* < 0.001). However, neither Gel 01 (Group C) nor VA5 (Group D) significantly increased the IgA levels in the lung and nasal wash compared with naked PRV antigen (*p* > 0.05). Meanwhile, the titers of IgA in nasal wash were lower than those in lung wash. These data demonstrate that the combination adjuvant comprising Gel 01 and VA5 is an effective delivery platform for the activation of the mucosal immune system.

### Cytokine responses *in vitro*

**FIGURE 4:**
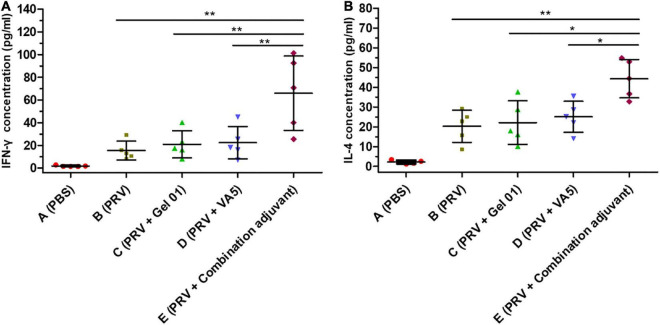
Cytokine responses induced by different antigen formulations. The concentrations (pg/ml) of IFN-γ **(A)** and IL-4 **(B)** in the supernatants of lymphocytes isolated from the spleens of mice (*n* = 5) were measured by ELISA at 42 dpi. The results are shown as the mean ± SD. The asterisks indicate significant differences (*p < 0.05, **p < 0.01).

To gain further understanding about the action mode of the combination adjuvant regarding the immune response, cytokine release was determined by evaluating Th1-type IFN-γ and Th2-type IL-4 concentrations in the splenocyte supernatant upon stimulation by inactive PRV (Figure 4 and Supplementary Table 4). As shown in Figures 4A,B, the combination adjuvant increased the concentrations of IFN-γ 4.29-fold, 3.14-fold, and 2.95-fold, and IL-4 2.18-fold, 2.00-fold, and 1.77-fold over naked PRV antigen, Gel 01 and VA5, respectively. The combination adjuvant significantly promoted the IFN-γ and IL-4 levels of the intranasal vaccine compared with naked PRV antigen, single Gel 01 adjuvant and single VA5 adjuvant (*p* < 0.05). However, neither Gel 01 nor VA5 significantly increased the concentrations of IFN-γ and IL-4 compared with naked PRV antigen (*p* > 0.05). These results indicated that the intranasal inactivated PRV vaccine formulated with the combination adjuvant effectively induced both Th1- and Th2-type immune responses.

### Influence of the combination adjuvant on the percentages of memory T lymphocytes in nasal-associated lymphoid tissue

**FIGURE 5:**
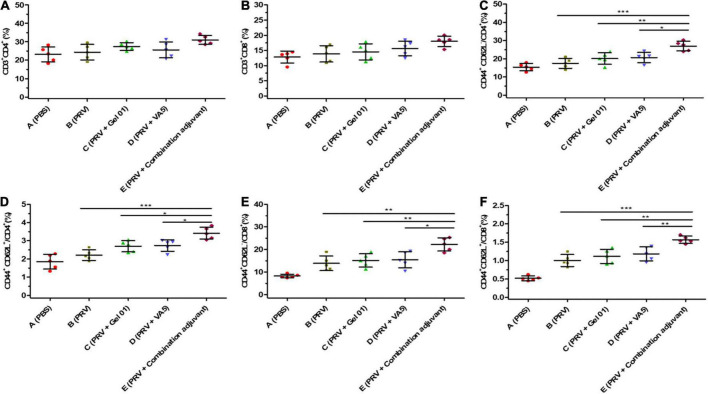
Mucosal immunization with the intranasal inactivated PR vaccine induces long-term T lymphocyte memory response. The percentages of CD3^+^CD4^+^ **(A)**, CD3^+^CD8^+^ **(B)**, CD44^+^ CD62L^–^/CD4^+^ **(C)**, CD44^+^CD62L^+^/CD4^+^ **(D)**, CD44^+^CD62L^–^/CD8^+^ **(E)**, and CD44^+^CD62L^+^/CD8^+^ **(F)** T lymphocytes from NALT at 42 dpi. CD3, CD4, CD8, CD62L, and CD44 positive cells were analyzed by flow cytometry. The CD44^+^CD62L^+^ central memory CD4^+^ and CD8^+^ T lymphocytes, as well as the CD44^+^CD62L^–^ effector memory CD4^+^ and CD8^+^ T lymphocytes, were analyzed in CD3^+^CD4^+^ and CD3^+^CD8^+^ T cells. Data represent the mean ± SD from 5 (*n*) mice per group. The asterisks indicate significant differences (*p < 0.05, **p < 0.01, ***p < 0.001).

Memory T cells play a critical role in the generation of protective immune responses, which are likely to be important in providing protection against virus infection (De Pelsmaeker et al., 2018; Wang et al., 2020b). Flow cytometry was performed to measure the percentages of CD3^+^CD4^+^, CD3^+^CD8^+^, CD44^+^CD62L^–^/CD4^+^, CD44^+^CD62L^+^/CD4^+^, CD44^+^CD62L^–^/CD8^+^, and CD44^+^CD62L^+^/CD8^+^ T lympho-cytes from mouse NALT at 42 dpi (Figure 5 and Supplementary Table 5). The percentages of CD3^+^CD4^+^ and CD3^+^CD8^+^ T lymphocyte subsets from Group E (PRV + Combination adjuvant) were slightly higher than those from Groups B (PRV), C (PRV + Gel 01) and D (PRV + VA5), but there was no significant difference among these immunized groups (*p* > 0.05). The percentages of effector memory T lymphocyte subsets CD44^+^CD62L^–^/CD4^+^ and CD44^+^CD62L^–^/CD8^+^ from Group E (PRV + Combination adjuvant) were significantly higher than those in Groups B (PRV), C (PRV + Gel 01) and D (PRV + VA5) (Figure 5) (*p* < 0.05). However, there was no significant difference in the percentages of effector memory T lymphocyte subsets CD44^+^CD62L^–^/CD4^+^ and CD44^+^CD62L^–^/CD8^+^ among Groups B, C, and D at 42 dpi (*p* > 0.05) (Figure 5). The percentages of the central memory T lymphocyte subsets CD44^+^CD62L^+^/CD4^+^ and CD44^+^CD62L^+^/CD8^+^ from Group E (PRV + Combination adjuvant) were significantly higher than those in the other immunized groups (Figure 5) (*p* < 0.05). There were no significant differences in the percentages of central memory T lymphocyte subsets CD44^+^CD62L^+^/CD4^+^ and CD44^+^CD62L^+^/CD8^+^ among Groups B, C, and D at 42 dpi (*p* > 0.05) (Figure 5). Collectively, the inactivated PRV antigen formulated with the combination adjuvant comprising Gel 01 and VA5 significantly promoted the proliferation of nasal memory T cells in mice compared to naked PRV antigen (Group B), Gel 01-adjuvanted PRV antigen (Group C) and VA5-adjuvanted PRV antigen (Group D) (*p* < 0.05). However, immunization with Gel 01-adjuvanted PRV antigen and VA5-adjuvanted PRV antigen did not significantly increase the proliferation of nasal memory T cells compared with immunization with naked PRV antigen (*p* > 0.05).

### Protective effect in mice immunized with the intranasal vaccine against pseudorabies virus challenge

**FIGURE 6:**
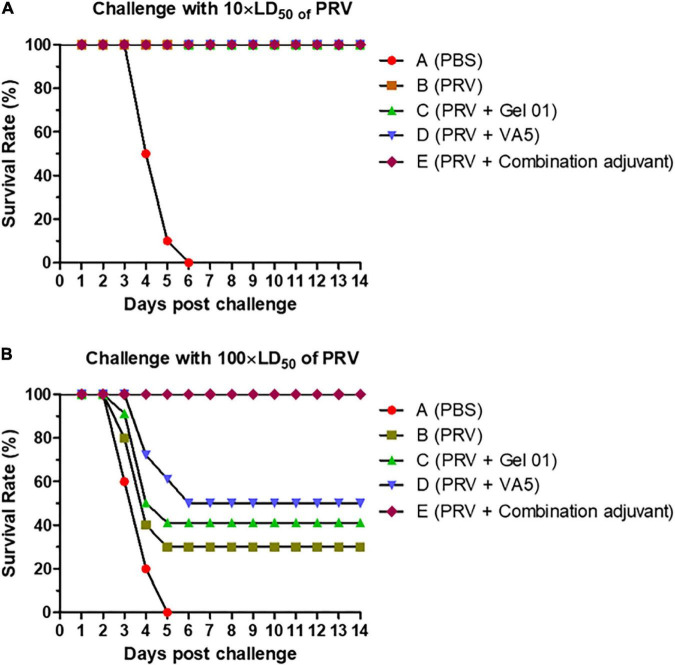
Survival rates of mice after the challenge with virulent pseudorabies virus (PRV). Percentage survival of mice (*n* = 10) after challenge with 10 × LD_50_ **(A)** or 100 × LD_50_ **(B)** of PRV. Mice were immunized intranasally with PBS **(A)**, naked PRV antigen (Group B), Gel 01 adjuvanted PRV antigen (Group C), VA5 adjuvanted PRV antigen (Group D) and antigen formulated in compound adjuvant (Group D). Mice were challenged intranasally with 10 × LD_50_ or 100 × LD_50_ of PRV at 42 dpi.

To evaluate the protective effect of the intranasally inactivated pseudorabies vaccine formulated with different adjuvants, ten mice from each group were challenged intranasally with 10 × LD_50_ or 100 × LD_50_ of virulent PRV at 42 dpi. The survival rates of these mice after this challenge are shown in Figure 6 and Supplementary Table 6. The protective effect of immunizing mice showed a dose-dependent challenge response. All the mice immunized intranasally with inactivated PRV vaccine, regardless of the use of Gel 01 or VA5 (Groups B– E), survived the intranasal challenge with 10 × LD_50_ of virulent PRV, while 100% of control mice (Group A) died after 6 days post challenge (dpc) (Figure 6A). Against to the intranasal challenge with 100 × LD_50_ of PRV, the survival percentages of mice immunized with naked PRV antigen (Group B), Gel 01 adjuvanted PRV antigen (Group C), VA5 adjuvanted PRV antigen (Group D) and antigen formulated with compound adjuvant (Group E) were 30, 40, 50, and 100%, respectively. None of the control mice vaccinated with PBS (Group A) survived the intranasal challenge with 100 × LD_50_ of virulent virus at 5 dpc. These results indicated that the protective efficacy of the intranasal inactivated PRV vaccine was effectively improved by the combination of Gel 01 and VA5 compared to naked PRV antigen, Gel 01-adjuvanted PRV antigen, and VA5-adjuvanted PRV antigen in mice.

## Discussion

Intramuscular vaccination with live attenuated and inactive PRV vaccines remains a widely used strategy to treat PRV infection and induce effective systemic protection (Zuckermann, 2000; Pomeranz et al., 2005). Unfortunately, intramuscular vaccination usually fails to induce local immunity in the upper respiratory mucosa, where tremendous numbers of PRV enter the pig body and can generate a “healthy carrier” state in pigs (Lipowski, 2006; Dong et al., 2014). In China, these “healthy carriers” of PRV caused severe antigenic mutation of the virus, resulting in the failure of the original vaccine immunity (An et al., 2013; Tong et al., 2015; Wang et al., 2016). Nasal vaccines have attracted increasing attention since they can evoke both mucosal and systemic immune responses (Shakya et al., 2016). Mucosal vaccination can induce sIgA antibodies, which play an important role in the prevention of respiratory illnesses. In addition, polymeric sIgA antibodies were proven to be strongly cross-reactive with other antigenic mutation strains (Renegar et al., 1998; Tamura et al., 2005). Intranasal vaccination with attenuated PRV vaccines can help to control PRV infection and dissemination in pigs (Ober et al., 1998; Dong et al., 2014; Salinas-Zacarias et al., 2020). However, in addition to pigs, PRV can infect a variety of other mammals, including bears, ruminants, carnivores and rodents, and it is fatal to these animals (Tan et al., 2021). Foxes infected with PRV led to the death of 1,200 animals (Jin et al., 2016) in Shandong Province, China. Similarly, minks infected with PRV caused the death of nearly 8,000 animals (Liu et al., 2017). In particular, recent studies have revealed that human beings might also be another potential host for PRV (Ai et al., 2018; Liu et al., 2021). To improve the safety of the intranasal PRV vaccine, inactivated PRV was used as an inoculate antigen in this study.

The inductive site for nasal immunity *in vivo* is the NALT (Sharma et al., 2009). The NALT comprises a cellular structure involved in the initiation and execution of an immune response, including dendritic cells, T cells and B cells, which are covered by an epithelial layer of microfold/membranous cells (or M cells) as well as the regional lymph from cervical lymph node which drains the NALT (Sharma et al., 2009). The transport of antigens across the epithelial barrier is a critical first step in the induction of immunity. The three barrier functions of the respiratory epithelium, which include mucociliary clearance, the maintenance of intercellular apical junctional complexes and the production of antimicrobial products of the airway, function together to effectively clear inhaled antigens (Wang et al., 2020a). Lone common immunogens deposited in the nasal cavity will be cleared with a half-life of approximately 15–20 min, and this is not enough time to generate a necessary immune response (Wu et al., 2012). M cells are present throughout the NALT epithelium and have variable microvilli/microfolds on their apical surfaces designed to facilitate the uptake of antigens, unlike surrounding epithelial cells (Man et al., 2004). Due to the lack of protein-degrading lysosomes in M cells, they do not have the ability to process antigens but instead transfer the captured antigens and IgA-antigen complexes to the underlying lymphoid cells (dendritic cells and B cells) for antigen processing and presentation (Komban et al., 2019; Huang et al., 2022). Some molecule immunopotentiators may penetrate the nasal epithelium and interact with underlying lymphoid cells. Because of the complexity of immune activation in NALT, inactivated antigens alone usually generate relatively weak immunity compared to live attenuated viruses (Li et al., 2020). Therefore, this study focused on developing new and effective mucosal adjuvants to improve the immunity efficacy of the intranasal inactivated PRV vaccine.

Mucoadhesive polymer can enhance the binding time of a vaccine antigen to the nasal mucosa and temporarily slow mucociliary clearance (Chaturvedi et al., 2011). Mucoadhesive polymers include polyacrylate, cellulose, chitosan, and gellan (Xu et al., 2021). Cross-linked polyacrylic polymers (Carbopol) can enhance the time of mucosal surface adhesion antigen and induce systemic antigen-specific IgG responses after intranasal vaccination (Coucke et al., 2009). MONTANIDE™ Gel 01 (SEPPIC, France) is based on the stable gel polymers of sodium polyacrylate in water, and it prolongs the residence time of the vaccine in the nasal mucosa (Deville et al., 2012; Li et al., 2019; Dessalegn et al., 2021). Pattern recognition receptor agonists have been extensively studied as mucosal adjuvants and can bind to pathogen recognition receptors on dendritic cells to activate downstream immune signals (Velasquez et al., 2010; Hjelm et al., 2014). These innate immune receptors are stimulated, along with the delivery of antigens to DCs, leading to Th1 responses and Th1-dependent antibody isotypes, and they can induce Th2 cytokines upon activation (Slutter et al., 2008). Mice nasally immunized with inactivated influenza virus adjuvanted with Poly I:C developed sIgA and systemic IgG antibodies, while parenteral delivery failed to elicit sIgA secretion in mice (Ichinohe et al., 2005). The addition of MDP to the inactivated respiratory syncytial virus promoted mucosal and systemic immunity in mice after respiratory tract immunization (Shafique et al., 2013). CVCVA5 (Chinese patent number 201210235427.0) is composed of the L–D isoform MDP (InvivoGen), poly I:C (InvivoGen), and levamisole hydrochloride (Sigma) in the aqueous phase (Wu et al., 2017). The VA5 adjuvant improved the serum and mucosal antibody response to avian influenza vaccine (Lu et al., 2016). However, there was no significant difference in immune response quality (including Th1 cytokines (IFN-γ), Th2 cytokines (IL-4), mucosal IgA, serum IgG and serum IgG2/IgG2 subtypes, etc.) among naked PRV antigen (Group B), Gel 01 adjuvanted PRV antigen (Group C), and VA5 adjuvanted PRV antigen (Group D) in this study (*p* > 0.05).

Due to the structural complexity of NALT, a single adjuvant sometimes cannot effectively improve the mucosal immune response and systemic response against antigens, and combination adjuvants have been extensively studied to overcome the ineffectiveness of a single adjuvant (Shakya et al., 2016). For example, the combination adjuvants AS04 and AS01B have been approved for use in humans by the FDA and have enhanced the immunogenicity of intranasal vaccines, as confirmed in various studies (Couch et al., 2009; Hirano et al., 2011; Blanco et al., 2014). Immunopotentiators (CpG-ODN or C48/80) were loaded into mucoadhesive polymer (chitosan) to form a combination adjuvant and were adopted to enhance the immunogenicity of the intranasal vaccine in mice (Li et al., 2021). The combination adjuvant comprised of polymer nanoparticles (dendrimer-like alpha-d-glucan) and Poly I:C can enhance the mucosal immune response and systemic responses to intranasal inactivated influenza vaccine in pigs (Renu et al., 2020). The combination adjuvant, composed of a polymer nanoemulsion (NE) and an RNA agonist of RIG-I (IVT DI), significantly improved the intranasal immune efficacy of the SARS-CoV-2 subunit vaccine in mice (Jangra et al., 2021). In this study, much stronger responses (Th1 cytokine (IFN-γ), Th2 cytokine (IL-4), mucosal IgA, serum IgG, and serum IgG2/IgG1 subtypes, etc.) were observed in the combination adjuvant comprising Gel 01 and VA5 compared to the single adjuvant. Therefore, our data clearly shows that there are synergistic effects between Gel 01 and VA5.

Polyacrylic acid (PAA) can form polyacrylate polymers with anionic constitutional units, which is a type of specific particulate delivery system (Zaman et al., 2010). Mucoadhesion occurs mainly through hydrogen bonding of polyacrylate polymers with mucin, a glycoprotein presents on the mucosal epithelium and M cells (Lele and Hoffman, 2000; Grabovac et al., 2005). When polyacrylate polymers contact the mucosal layer on nasal epithelium, they become hydrated, swell, bind to the mucus layer (Chaturvedi et al., 2011; Xu et al., 2021). Mucoadhesives can temporarily inhibit ciliary movement and prolong the residence time of the vaccine antigen in the nasal mucosa, and finally promote the capture of antigen by M cells (Chaturvedi et al., 2011). It has been suggested that PAA-based formulations not only enhance the adhesive time of formulations but also promote permeation of the tight junctions between epithelial cells (Lele and Hoffman, 2000; Ugwoke et al., 2005). Promoted paracellular transport induced by PAA can be found in its effect on the tight junctions of epithelial cells by the PAA binding to extracellular Ca^2 +^, therefore reducing its concentration, which directly affects the permeability of epithelial tight junctions (Lele and Hoffman, 2000; Ugwoke et al., 2005). In addition, the water absorption from mucus by polyacrylate polymers dries epithelial cells causing the tight junctions to separate, so that absorption increases *via* the paracellular pathway (Coucke et al., 2009). These effects were found to be successful in opening epithelial tight junctions and establishing paracellular transport. Not only PAA but also its commercial derivatives (Carbopol^®^ and Carbomer^®^) exhibit strong mucoadhesion and absorption-promotion (Gasper et al., 2016; Chen et al., 2019; Correia et al., 2022). MONTANIDE™ Gel 01 is a hydrogel polymer of sodium polyacrylate, which is also a commercial derivative of PAA. The aqueous components of VA5 adjuvant are based on ligands to pattern recognition receptors, poly I:C, MDP, and an immune enhancement chemical, levamisole hydrochloride, in PBS. Poly I:C has been shown to enhance the activity of respiratory dendritic cells and helps the migration of both effector and memory T cells (McNally et al., 2012; Perez-Giron et al., 2014). Levamisole promoted murine bone marrow-derived dendritic cell activation and drove the Th1 immune response *in vitro* and *in vivo* (Fu et al., 2016). MDP induces autophagy in dendritic cells influencing antigen presentation (Cooney et al., 2010). In this study, the results indicated that the combination adjuvant composed of Gel 01 and VA5 efficiently boosted immune responses to intranasal inactivated PR vaccine compared with single Gel 01 or VA5 adjuvanted antigen. We speculated that Gel 01 adjuvant alone could only improve the temporary retention time of the antigen in the nasal mucosa. Gel 01 combined with VA5 adjuvant not only prolonged the retention time of antigen in nasal mucosa but also improved the bioavailability and action time of immune-modulatory compounds (VA5). VA5 might further improve the immunostimulatory potential of antigens and the recruitment of the innate immune system, in turn resulting in the downstream activation of adaptive immunity.

Once antigens are transported through the mucosa of the upper respiratory tract, they initiate the immune response in inductive sites (such as NALT) and then induce both local mucosal immune responses and systemic immune responses (Holmgren and Czerkinsky, 2005). sIgA is the major antibody isotype in mucosal immunity (Asahi-Ozaki et al., 2004). IgA antibodies, which have higher avidity than IgG antibodies, can readily access mucosal viral antigens and provide protection against heterologous strains (Muramatsu et al., 2014). Intranasal vaccine administration induced higher secretory IgA production than administration by the parenteral route. Intranasal administration of a bivalent inactivated influenza virus vaccine along with poly (I:C) induced high IgA levels and protected mice from heterologous strains (Ichinohe et al., 2007). It has long been recognized in both humans and animal models that memory CD8^+^ T lymphocytes play an important role in cross-protection against antigenic variants and heterologous virus strains (Kappes et al., 2012; Zens et al., 2016; Pizzolla et al., 2018; Koutsakos et al., 2019). Intranasal immunization with inactivated H1N1 vaccine formulated with the combination adjuvant (PS-GAMP) elevated IgA titers and memory CD8^+^ T cells, and conferred strong cross-protection against lethal challenges with distant H1N1 and heterosubtypic H3N2, H5N1, and H7N9 viruses for at least 6 months in mice (Wang et al., 2020b). In this study, our data indicated that the combination adjuvant of Gel 01 and VA5 efficiently boosted mucosal IgA against PRV and the central and effector memory subsets of CD8^+^ T cells compared with naked inactivated antigen, single Gel 01 or VA5 adjuvanted antigen. Therefore, intranasal vaccines formulated with the combination adjuvant may offer cross-protection against the novel antigenic variant PRV, which needs to be further studied.

It has been demonstrated that the proliferation of CD8^+^ and CD4^+^CD8^+^ PRV-experienced T cells might directly contribute to the elimination of PRV-infected cells (De Pelsmaeker et al., 2018). Memory CD8^+^ T lymphocytes have been found to play an important role in protective immune responses against viral infection (Wang et al., 2020b). Intranasal immunization with recombinant Tm-WAP49 protein formulated with the combination adjuvant of OCH (the irritant of natural killer T cells) and QS-21 (*Quillaja saponaria*) increased the central (CD62L^+^CD44^+^) and effector (CD62L^–^CD44^+^) memory subsets of both CD4^+^ and CD8^+^ T cells and induced strong protection against murine trichuriasis (Wei et al., 2022). Immune memory is a key issue for the success of a vaccine to provide extended, adequate, and rapid protection against pathogens (Stern, 2020). In this study, our results demonstrated that mice immunized intranasally with inactivated PRV formulated with a combination of Gel 01 and VA5 could promote the proportions of the central (CD62L^+^CD44^+^) and effector (CD62L^–^CD44^+^) memory subsets of both CD4^+^ and CD8^+^ T cells. The protective effect against PRV was dependent on the vaccine and challenge virus doses. Here, mice vaccinated intranasally with inactivated PRV antigen with or without adjuvant showed 100% protection against challenge with a low dose of 10 × LD_50_ live PRV. Against the challenge with a high dose of 100 × LD_50_ of virus, the protective efficacy of intranasal PRV vaccination was effectively improved by the combination adjuvant of Gel 01 and VA5 compared with other vaccines in mice.

In summary, intranasal immunization with inactivated PRV antigen, formulated with the combination adjuvant of Gel 01 and VA5, induced higher mucosal immunity (sIgA) and systemic immune responses (IgG, IgG1, and IgG2a) than naked PRV antigen, Gel 01-adjuvanted PRV antigen and VA5-adjuvanted PRV antigen. In addition, the *in vitro* cytokine assay exhibited mixed Th1/Th2 responses, suggesting an enhanced and mixed humoral/cellular immune response by antigens co-delivered with the combination adjuvant of Gel 01 and VA5. In addition, inactivated PRV antigen formulated with a combination of Gel 01 and VA5 was found to stimulate increased proportions of the central (CD62L^+^CD44^+^) and effector (CD62L^–^CD44^+^) memory subsets of both CD4^+^ and CD8^+^ T cells in the NALT; this conferred significant protection in immunized mice against intranasal challenge with a high dose of 100 × LD_50_ of PRV and might provide strong cross-protection for future allogenetic PRV. The results obtained in this study suggest that mucosal immunity with intranasal PR vaccine formulated with the combination adjuvant of Gel 01 and VA5 is an effective vaccination approach to induce protective immunity against PRV.

## Data availability statement

The original contributions presented in this study are included in the article/Supplementary material, further inquiries can be directed to the corresponding author.

## Ethics statement

The study and protocol were both approved by the Science and Technology Agency of Jiangsu Province (approval number: NKYVET 2015-0066) and by the Jiangsu Academy of Agricultural Sciences Experimental Animal Ethics Committee.

## Author contributions

TH, DZ, and BT conceived and designed the experiments. TH, CC, XZ, YH, HW, DZ, and BT performed the experiments. TH, CC, DZ, and BT analyzed the data. TH, CC, HW, and BT contributed reagents, materials, and analysis tools and wrote and editing the manuscript. All authors contributed to the article and approved the submitted version.
