# Supplementary material for: Protective efficacy of intranasal inactivated pseudorabies vaccine is improved by combination adjuvant in mice
Source: Front Microbiol. 2022 Sep 15;13:976220. doi: 10.3389/fmicb.2022.976220 (PMC9520748; doi:10.3389/fmicb.2022.976220)

# Protective Efficacy of Intranasal Inactivated Pseudorabies Vaccine is Improved by Combination Adjuvant in Mice

Tao Hua^1, 2, 3, 4^, Chen Chang^1, 2, 3, 4^, Xuehua Zhang^1, 2, 3, 4^, Yuqing Huang^1, 2, 3, 4^, Haiyan Wang^1, 2, 3, 4^, Daohua Zhang^1, 2, 3, 4^, Bo Tang^1, 2, 3, 4^*

^1^Institute of Veterinary Immunology & Engineering, Jiangsu Academy of Agricultural Sciences, Nanjing, China; ^2^National Research Center of Veterinary Bio-product Engineering and Technology, Jiangsu Academy of Agricultural Science, Nanjing, China; ^3^Jiangsu Key Laboratory for Food Quality and Safety-State Key Laboratory Cultivation Base, Ministry of Science and Technology, Nanjing, China; ^4^Jiangsu Co-innovation Center for Prevention and Control of Important Animal Infectious Diseases and Zoonoses, Yangzhou, China

***Corresponding author:**

Bo Tang

Institute of Veterinary Immunology & Engineering

Jiangsu Academy of Agricultural Science, Nanjing 210014, P.R. China

Tel.: 86 25 84392019

Fax: 86 25 84392028

Email: tangbojaas@sina.com

**Data Sheet:**

| Group (vaccine) | Days post first immunization | PRV-specific antibodies from mice serum (n=10) were measured by ELISA.^α^ | | | | | | | | | | mean ± SD |
| --- | --- | --- | --- | --- | --- | --- | --- | --- | --- | --- | --- | --- |
|  |  | 1 | 2 | 3 | 4 | 5 | 6 | 7 | 8 | 9 | 10 |  |
| A (PBS) | 21 | 0 | 0 | 0 | 0 | 0 | 0 | 0 | 0 | 0 | 0 | 0 |
|  | 42 | 0 | 0 | 0 | 0 | 0 | 0 | 0 | 0 | 0 | 0 | 0 |
| B (PRV) | 21 | 100 | 400 | 100 | 200 | 200 | 200 | 400 | 200 | 200 | 200 | 220 ± 103 |
|  | 42 | 400 | 1600 | 400 | 800 | 800 | 800 | 1600 | 800 | 800 | 800 | 880 ± 413 |
| C (PRV + Gel 01) | 21 | 200 | 400 | 400 | 200 | 200 | 400 | 200 | 200 | 200 | 200 | 260 ± 96 |
|  | 42 | 800 | 1600 | 1600 | 800 | 800 | 1600 | 800 | 1600 | 800 | 800 | 1120± 413 |
| D (PRV + VA5) | 21 | 100 | 400 | 400 | 400 | 400 | 400 | 400 | 200 | 200 | 200 | 310 ± 119 |
|  | 42 | 400 | 1600 | 800 | 1600 | 1600 | 1600 | 1600 | 800 | 800 | 800 | 1160 ± 478 |
| E (PRV + Combination adjuvant) | 21 | 800 | 400 | 800 | 800 | 400 | 800 | 400 | 800 | 400 | 800 | 640 ± 206 |
|  | 42 | 6400 | 1600 | 3200 | 3200 | 1600 | 3200 | 1600 | 3200 | 1600 | 3200 | 2880 ± 1470 |

Supplemental Table 1. PRV-specific IgG titers from mouse serum measured by ELISA at 21 and 42 days post-first immunization (n=10).

^α^ PRV-specific antibody titer was considered to be negative when titers were scored below 100-fold dilution.

Supplemental Table 2. Antigen-specific IgG2a and IgG1 titers from mouse serum at 42 days post-first immunization (n=10).

| Group (vaccine) | Mouse No. | IgG2a antibody titers^α^ | IgG1 antibody titers^α^ | IgG2a/IgG1 ratio |
| --- | --- | --- | --- | --- |
| A (PBS) | 1 | 0 | 0 |  |
|  | 2 | 0 | 0 |  |
|  | 3 | 0 | 0 |  |
|  | 4 | 0 | 0 |  |
|  | 5 | 0 | 0 |  |
|  | 6 | 0 | 0 |  |
|  | 7 | 0 | 0 |  |
|  | 8 | 0 | 0 |  |
|  | 9 | 0 | 0 |  |
|  | 10 | 0 | 0 |  |
|  | mean ± SD | 0 | 0 |  |
| B (PRV) | 1 | 100 | 400 | 0.25 |
|  | 2 | 200 | 1600 | 0.125 |
|  | 3 | 100 | 400 | 0.25 |
|  | 4 | 200 | 800 | 0.25 |
|  | 5 | 100 | 800 | 0.125 |
|  | 6 | 100 | 800 | 0.125 |
|  | 7 | 400 | 1600 | 0.25 |
|  | 8 | 100 | 800 | 0.125 |
|  | 9 | 100 | 400 | 0.25 |
|  | 10 | 100 | 800 | 0.125 |
|  | mean ± SD | 150 ± 97 | 840 ± 440 | 0.187 ± 0.065 |
| C (PRV + Gel 01) | 1 | 200 | 800 | 0.25 |
|  | 2 | 200 | 1600 | 0.125 |
|  | 3 | 400 | 1600 | 0.25 |
|  | 4 | 100 | 800 | 0.125 |
|  | 5 | 100 | 400 | 0.25 |
|  | 6 | 400 | 1600 | 0.25 |
|  | 7 | 200 | 800 | 0.25 |
|  | 8 | 200 | 1600 | 0.125 |
|  | 9 | 200 | 800 | 0. 25 |
|  | 10 | 100 | 800 | 0.125 |
|  | mean ± SD | 210 ± 110 | 1080 ± 463 | 0.200 ± 0.064 |
| D (PRV + VA5) | 1 | 100 | 400 | 0.25 |
|  | 2 | 400 | 1600 | 0.25 |
|  | 3 | 200 | 800 | 0.25 |
|  | 4 | 400 | 1600 | 0.25 |
|  | 5 | 200 | 1600 | 0.125 |
|  | 6 | 400 | 1600 | 0.25 |
|  | 7 | 400 | 1600 | 0.25 |
|  | 8 | 200 | 800 | 0.25 |
|  | 9 | 200 | 800 | 0.25 |
|  | 10 | 200 | 800 | 0.25 |
|  | mean ± SD | 270 ± 115 | 1160 ± 478 | 0.237 ± 0.039 |
| E (PRV + Combination adjuvant) | 1 | 1600 | 3200 | 0.5 |
|  | 2 | 800 | 1600 | 0.5 |
|  | 3 | 800 | 3200 | 0.25 |
|  | 4 | 1600 | 3200 | 0.5 |
|  | 5 | 800 | 1600 | 0.5 |
|  | 6 | 800 | 3200 | 0.25 |
|  | 7 | 400 | 1600 | 0.25 |
|  | 8 | 800 | 1600 | 0.5 |
|  | 9 | 400 | 1600 | 0.25 |
|  | 10 | 800 | 3200 | 0.25 |
|  | mean ± SD | 880 ± 413 | 2400 ± 843 | 0.375 ± 0.131 |

^α^ Antigen-specific IgG2a and IgG1 titers were considered to be negative when titers were scored below 100-fold dilution.

Supplemental Table 3. Mucosa IgA titers in lung wash and nasal wash at 42 days post-first immunization (n=5).

| Group (vaccine) | Mouse No. | lung wash IgA titers^α^ | Nasal wash IgA titers^α^ |
| --- | --- | --- | --- |
| A (PBS) | 1 | 0 | 0 |
|  | 2 | 0 | 0 |
|  | 3 | 0 | 0 |
|  | 4 | 0 | 0 |
|  | 5 | 0 | 0 |
|  | mean ± SD | 0 | 0 |
| B (PRV) | 1 | 80 | 20 |
|  | 2 | 320 | 80 |
|  | 3 | 80 | 20 |
|  | 4 | 160 | 40 |
|  | 5 | 160 | 40 |
|  | mean ± SD | 160 ± 97 | 40 ± 24 |
| C (PRV + Gel 01) | 1 | 80 | 20 |
|  | 2 | 320 | 80 |
|  | 3 | 160 | 40 |
|  | 4 | 160 | 40 |
|  | 5 | 160 | 40 |
|  | mean ± SD | 176 ± 87 | 44 ± 21 |
| D (PRV + VA5) | 1 | 80 | 20 |
|  | 2 | 320 | 80 |
|  | 3 | 160 | 40 |
|  | 4 | 160 | 20 |
|  | 5 | 320 | 80 |
|  | mean ± SD | 208 ± 107 | 48 ± 30 |
| E (PRV + Combination adjuvant) | 1 | 640 | 160 |
|  | 2 | 320 | 80 |
|  | 3 | 640 | 160 |
|  | 4 | 640 | 160 |
|  | 5 | 320 | 80 |
|  | mean ± SD | 512 ± 175 | 128 ± 43 |

^α^ Mucosa IgA titers were considered to be negative when titers were scored below 20-fold dilution.

Supplemental Table 4. The concentrations (pg/ml) of IFN-γ and IL-4 in the supernatants of lymphocytes isolated from the spleens of mice (n=5) were measured by ELISA 42 days after the first nasal immunization.

| Group (vaccine) | Mouse No. | IFN-γ concentration (pg/ml) | IL-4 concentration (pg/ml) |
| --- | --- | --- | --- |
| A (PBS) | 1 | 1.63 | 2.03 |
|  | 2 | 2.72 | 1.15 |
|  | 3 | 1.52 | 3.41 |
|  | 4 | 1.43 | 2.55 |
|  | 5 | 1.86 | 1.55 |
|  | mean ± SD | 1.83 ± 0.52 | 2.13 ± 0.88 |
| B (PRV) | 1 | 8.12 | 8.62 |
|  | 2 | 29.23 | 29.12 |
|  | 3 | 10.56 | 15.68 |
|  | 4 | 15.87 | 25.08 |
|  | 5 | 13.02 | 22.82 |
|  | mean ± SD | 15.36 ± 8.26 | 20.26 ± 8.13 |
| C (PRV + Gel 01) | 1 | 17.56 | 17.9 |
|  | 2 | 40.04 | 37.68 |
|  | 3 | 23.04 | 28.82 |
|  | 4 | 16.05 | 16.08 |
|  | 5 | 8.05 | 10.08 |
|  | mean ± SD | 20.94 ± 11.94 | 22.11 ± 11.02 |
| D (PRV + VA5) | 1 | 7.06 | 14.08 |
|  | 2 | 45.04 | 35.5 |
|  | 3 | 16.04 | 22.1 |
|  | 4 | 18.04 | 25.1 |
|  | 5 | 25.45 | 28.5 |
|  | mean ± SD | 22.32 ± 14.28 | 25.05 ± 7.90 |
| E (PRV + Combination adjuvant) | 1 | 101.25 | 54.82 |
|  | 2 | 25.45 | 32.82 |
|  | 3 | 92.36 | 52.9 |
|  | 4 | 70.68 | 44.48 |
|  | 5 | 40.02 | 36.7 |
|  | mean ± SD | 65.95 ± 32.70 | 44.34 ± 9.67 |

Supplemental Table 5. The percentages of CD3^+^CD4^+^, CD3^+^CD8^+^, CD44^+^CD62L^-^/CD4^+^, CD44^+^CD62L^+^/CD4^+^, CD44^+^CD62L^-^/CD8^+^, and CD44^+^CD62L^+^/CD8^+^ T lymphocytes from NALT at 42 days post-first immunization.

| Group (vaccine) | Mouse No. | CD3^+^CD4^+^ (%) | CD3^+^CD8^+^ (%) | CD44^+^CD62L^-^/CD4^+^(%) | CD44^+^CD62L^+^/CD4^+^(%) | CD44^+^CD62L^-^/CD8^+^(%) | CD44^+^CD62L^+^/CD8^+^(%) |
| --- | --- | --- | --- | --- | --- | --- | --- |
| A (PBS) | 1 | 23.45 | 12.5 | 15.5 | 1.88 | 7.92 | 0.48 |
|  | 2 | 28.21 | 14.45 | 17.92 | 2.28 | 9.5 | 0.62 |
|  | 3 | 18.25 | 9.52 | 12.62 | 1.55 | 7.84 | 0.45 |
|  | 4 | 20.14 | 13.62 | 14.52 | 1.33 | 7.45 | 0.53 |
|  | 5 | 25.74 | 13.92 | 16.45 | 2.2 | 8.62 | 0.51 |
|  | mean ± SD | 23.15 ± 4.04 | 12.80 ± 1.96 | 15.40 ± 1.99 | 1.84 ± 0.40 | 8.26 ± 0.80 | 0.51 ± 0.06 |
| B (PRV) | 1 | 19.08 | 10.85 | 15.85 | 1.88 | 10.85 | 0.82 |
|  | 2 | 29.58 | 16.95 | 21.05 | 2.63 | 18.95 | 1.25 |
|  | 3 | 21.55 | 11.52 | 14.02 | 2.05 | 11.52 | 0.93 |
|  | 4 | 24.25 | 14.02 | 17.52 | 2.15 | 13.02 | 0.95 |
|  | 5 | 27.25 | 16.05 | 18.95 | 2.33 | 15.05 | 1.06 |
|  | mean ± SD | 24.34 ± 4.22 | 13.87 ± 2.68 | 17.47 ± 2.71 | 2.20 ± 0.28 | 13.87 ± 3.26 | 1.00 ± 0.16 |
| C (PRV + Gel 01) | 1 | 27.28 | 16.03 | 22.04 | 2.8 | 15.04 | 1.12 |
|  | 2 | 28.54 | 15.04 | 20.03 | 2.88 | 17.03 | 1.26 |
|  | 3 | 30.25 | 17.84 | 23.84 | 3.05 | 18.84 | 1.35 |
|  | 4 | 26.45 | 12.47 | 19.47 | 2.4 | 13.47 | 0.94 |
|  | 5 | 24.75 | 11.31 | 15.31 | 2.35 | 11.31 | 0.91 |
|  | mean ± SD | 27.45 ± 2.08 | 14.53 ± 2.64 | 20.33 ± 3.51 | 2.69 ± 0.30 | 15.13 ± 2.94 | 1.11 ± 0.19 |
| D (PRV + VA5) | 1 | 20.74 | 12.45 | 16.45 | 2.23 | 10.45 | 0.95 |
|  | 2 | 31.25 | 18.43 | 24.25 | 3.01 | 18.43 | 1.35 |
|  | 3 | 25.5 | 16.17 | 21.17 | 2.93 | 15.17 | 1.18 |
|  | 4 | 28.47 | 17.25 | 21.43 | 2.88 | 19.25 | 1.39 |
|  | 5 | 22.25 | 13.93 | 19.93 | 2.63 | 13.93 | 1.05 |
|  | mean ± SD | 25.64 ± 4.33 | 15.44 ± 2.42 | 20.64 ± 2.82 | 2.73 ± 0.31 | 15.44 ± 3.56 | 1.18 ± 0.18 |
| E (PRV + Combination adjuvant) | 1 | 34.25 | 20.02 | 30.32 | 3.83 | 25.32 | 1.7 |
|  | 2 | 28.55 | 15.32 | 24.55 | 3.05 | 18.55 | 1.53 |
|  | 3 | 32.58 | 18.55 | 28.43 | 3.38 | 20.02 | 1.54 |
|  | 4 | 30.44 | 18.43 | 27.65 | 3.65 | 24.65 | 1.62 |
|  | 5 | 29.25 | 17.65 | 24.02 | 3.15 | 22.43 | 1.45 |
|  | mean ± SD | 31.01 ± 2.36 | 17.99 ± 1.72 | 26.99 ± 2.66 | 3.41 ± 0.32 | 22.19 ± 2.90 | 1.56 ± 0.09 |

Supplemental Table 6. Survival rates of mice after the challenge with virulent pseudorabies virus.

| Group (vaccine) | Challenge dose^α^ | Deaths/survivals at day post challenge | | | | | | | | | | | | | | Survival rates |
| --- | --- | --- | --- | --- | --- | --- | --- | --- | --- | --- | --- | --- | --- | --- | --- | --- |
|  |  | 1 | 2 | 3 | 4 | 5 | 6 | 7 | 8 | 9 | 10 | 11 | 12 | 13 | 14 |  |
| A (PBS) | 10×LD_50_ | 0/10 | 0/10 | 0/10 | 5/5 | 4/1 | 1/0 | 0/0 | 0/0 | 0/0 | 0/0 | 0/0 | 0/0 | 0/0 | 0/0 | 0% |
|  | 100×LD_50_ | 0/10 | 0/10 | 4/6 | 4/2 | 2/0 | 0/0 | 0/0 | 0/0 | 0/0 | 0/0 | 0/0 | 0/0 | 0/0 | 0/0 | 0% |
| B (PRV) | 10×LD_50_ | 0/10 | 0/10 | 0/10 | 0/10 | 0/10 | 0/10 | 0/10 | 0/10 | 0/10 | 0/10 | 0/10 | 0/10 | 0/10 | 0/10 | 100% |
|  | 100×LD_50_ | 0/10 | 0/10 | 2/8 | 4/4 | 1/3 | 0/3 | 0/3 | 0/3 | 0/3 | 0/3 | 0/3 | 0/3 | 0/3 | 0/3 | 30% |
| C (PRV + Gel 01) | 10×LD_50_ | 0/10 | 0/10 | 0/10 | 0/10 | 0/10 | 0/10 | 0/10 | 0/10 | 0/10 | 0/10 | 0/10 | 0/10 | 0/10 | 0/10 | 100% |
|  | 100×LD_50_ | 0/10 | 0/10 | 1/9 | 4/5 | 1/4 | 0/4 | 0/4 | 0/4 | 0/4 | 0/4 | 0/4 | 0/4 | 0/4 | 0/4 | 40% |
| D (PRV + VA5) | 10×LD_50_ | 0/10 | 0/10 | 0/10 | 0/10 | 0/10 | 0/10 | 0/10 | 0/10 | 0/10 | 0/10 | 0/10 | 0/10 | 0/10 | 0/10 | 100% |
|  | 100×LD_50_ | 0/10 | 0/10 | 0/10 | 3/7 | 1/6 | 1/5 | 0/5 | 0/5 | 0/5 | 0/5 | 0/5 | 0/5 | 0/5 | 0/5 | 50% |
| E (PRV + Combination adjuvant) | 10×LD_50_ | 0/10 | 0/10 | 0/10 | 0/10 | 0/10 | 0/10 | 0/10 | 0/10 | 0/10 | 0/10 | 0/10 | 0/10 | 0/10 | 0/10 | 100% |
|  | 100×LD_50_ | 0/10 | 0/10 | 0/10 | 0/10 | 0/10 | 0/10 | 0/10 | 0/10 | 0/10 | 0/10 | 0/10 | 0/10 | 0/10 | 0/10 | 100% |

^α^Mice were challenged intranasally with 10×LD_50_ or 100×LD_50_ of PRV 42 days after the first immunization.

Supplemental Figure 1. The Chinese patent 201210235427.0, which describe the protocol to develop the CVCVA5 adjuvant including the concentration of each component and the procedure when mixed with antigen.


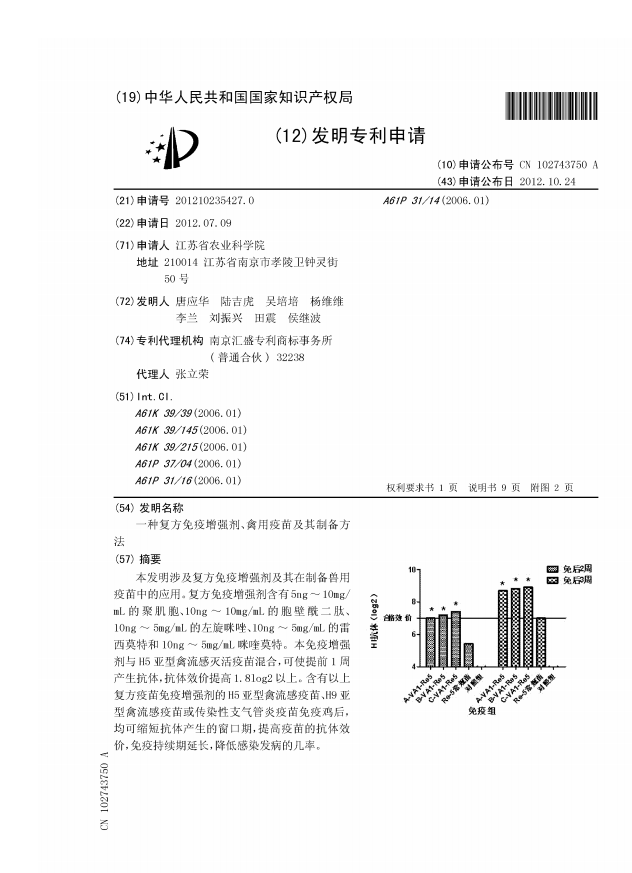

Supplement: Supplementary file 1 [file Data_Sheet_1.DOC]
